# Enhancing the Understanding of Aroma Formation during Coffee Roasting Using DHS-GC×GC-TOFMS and Chemometrics

**DOI:** 10.1021/acsomega.5c01783

**Published:** 2025-06-13

**Authors:** Andre Cunha Paiva, Carlos Alberto Teixeira, Leandro Wang Hantao

**Affiliations:** † Instituto de Química, Universidade Estadual de Campinas, 270 Monteiro Lobato, Campinas, São Paulo 13083-862, Brasil; ‡ Instituto Nacional de Ciência e Tecnologia (INCTBio), Campinas, São Paulo 13083-862, Brasil

## Abstract

This study integrates advanced chromatographic techniques and chemometric analysis to deepen the understanding of volatile and semivolatile compound formation during coffee roasting. Dynamic headspace extraction (DHS) was used to capture these compounds at three distinct roasting stages. The volatiles were analyzed and identified using comprehensive two-dimensional gas chromatography coupled with time-of-flight mass spectrometry (GC×GC-TOFMS), a high-resolving power technique to reliably annotate the numerous features present in the aroma-profile. Partial least-squares discriminant analysis (PLS-DA) was used to handle the large and complex data generated by GC×GC-TOFMS. This method successfully classified samples based on the roasting stages. Evaluation of the loadings using a variable importance in projection (VIP) approach revealed that 99 aroma-related volatiles varied significantly during coffee roasting, including carboxylic acids (e.g., acetic acid) and pyrazines such as 2-ethenyl-5-methyl pyrazine and ethyl pyrazine. These compounds exhibited distinct kinetic profiles, providing a detailed understanding of the chemical transformations occurring during roasting. This research describes an important methodology for monitoring aroma-related volatiles that may be useful for personalized coffee roasting, delivering key insights into the complex development of coffee aroma.

## Graphical abstract

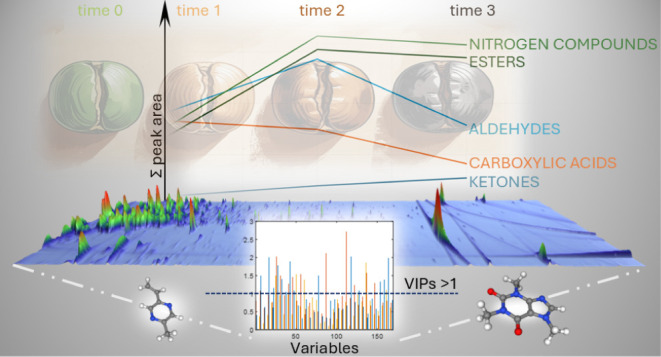

## 1 Introduction

Coffee is celebrated not only for its stimulating properties but also for its captivating aroma, which develops during the roasting process.^1,2^ Research into coffee’s aroma dates to the early 20th century, when researchers began identifying over 29 volatile compounds in roasted coffee.^1,2^ Since then, advancements in gas chromatography (GC) and mass spectrometry (MS) have revealed that both green and roasted coffee contain over 1,000 volatile and semivolatile organic compounds (VOCs and SVOCs),^3^ each contributing to its complex aroma profile. As the catalog of these compounds expands, researchers have shifted their focus to determining which of these are sensorially active and how they influence the aroma of roasted coffee.^1–3^

Home coffee roasters provide enthusiasts with the unique opportunity to personalize the aroma profile of their coffee by experimenting with different roasting techniques and parameters. Unlike prepackaged commercial coffee, which often offers a fixed flavor profile, roasting at home allows for precise control over key variables such as temperature, roasting time, and air flow, all of which significantly impact the development of aromatic compounds.^2^ By adjusting these factors, users can influence the formation of specific volatile and semivolatile organic compounds. For instance, a lighter roast might highlight floral and fruity notes due to the preservation of delicate compounds like linalool and aldehydes, while a darker roast can enhance the presence of chocolatey or nutty aromas through the increased formation of Maillard reaction products such as pyrazines and furans. Additionally, home roasters can experiment with different green coffee varieties, blending techniques, and roast profiles, enabling them to tailor the aroma and flavor to their exact preferences.

While much of the research has focused on roasted coffee, the composition of VOCs in raw coffee beans has also gained attention.^4^ Despite being present in lower concentrations, green coffee VOCs provide valuable insights for identifying potential off-flavors before roasting and predicting the final aroma quality.^4^ Roasting induces the formation of key odoriferous compounds, including aldehydes, pyrazines, phenols, and thiols, which are responsible for the distinctive aromas associated with coffee. The roasting process itself involves exposing raw coffee beans to high temperatures, triggering intricate chemical and physical changes such as moisture loss and density reduction.^2^

The volatile profile of roasted coffee is intricate, with numerous VOCs and SVOCs that interact to create its characteristic aroma. Although extensive studies have been conducted on the chemical transformations during roasting and the design of roasting equipment,^1,2,5–10^ the field remains far from fully understood.^2^ Several variables influence the formation of these aroma compounds, including the time and temperature profiles used, the unique properties of the beans, and cultivation and processing techniques.^3^ Thus, the roasting process is often considered a craft or an art.^4^

To develop a scientific foundation for optimizing coffee aroma, researchers have explored various analytical techniques to monitor the evolution of these compounds during roasting. Some studies have correlated specific VOCs concentrations with sensory analyses,^4^ while others have used model systems to simulate the chemical transformations occurring during roasting.^1,2^ However, findings from these simplified models must be interpreted with caution, as they may not capture the complexity of reactions in real coffee beans.^4^ A more elucidative approach to study the transformations occurring in raw coffee during roasting can be achieved by analyzing the volatile plume released throughout the actual roasting process.^10^

Traditional extraction techniques like solid-phase microextraction (SPME) are commonly used to profile VOCs in food matrices.^11^ However, dynamic headspace (DHS) extraction offers a significant advantage for monitoring the evolution of coffee aroma during roasting. In DHS, the volatile and semivolatile compounds are continuously captured on a sorbent tube, providing higher extraction capacity and sensitivity compared to SPME.^12^ These compounds are then thermally desorbed and analyzed using gas chromatography, making DHS ideal for real-time profiling.

Despite the use of conventional one-dimensional gas chromatography (1D-GC) in analyzing coffee,^5,6,8,9,13^ it often struggles with peak overlap due to the complex nature of the sample. Comprehensive two-dimensional gas chromatography coupled with mass spectrometry (GC×GC-MS) addresses this limitation by providing enhanced peak separation and a more detailed characterization of the coffee matrix. While some approaches have tried to monitor roasting without GC,^7,14–16^ these methods face challenges in compound identification, as condensed spectral data cannot be easily deconvoluted. In contrast, using GC×GC with electron ionization and commercial MS libraries such as NIST improves the reliability of analyte identification.^17^ Given the vast amount of data generated by DHS-GC×GC-MS, manual analysis of each chromatographic peak would be inefficient and could lead to the loss of critical information. Therefore, chemometric tools are necessary for effective data mining.

In this study, the main objective is to develop an analytical setup for monitoring the volatile profile during the coffee bean roasting process. In this scenario, a DHS-GC×GC-TOFMS method was evaluated for monitoring aroma-related volatiles produced during coffee roasting, which may provide important insights into the complex development of coffee aroma. Sampling of aroma-related volatiles was performed during three distinct roasting stages. Using a custom-built lab roaster, five subgroups of *Coffea arabica* were analyzed. VOCs and SVOCs were captured using DHS and analyzed by GC×GC-TOFMS. Partial least-squares discriminant analysis (PLS-DA) successfully differentiated the samples across the roasting stages, while the Variable Importance in Projection (VIP) approach identified key compounds with significant concentration variations. This integrated methodology provided a powerful tool for real-time profiling of aroma compounds, contributing with valuable insights into coffee roasting chemistry and supporting the development of optimized roasting strategies.

## 2 Material and Methods

### 2.1 Samples

Five batches containing 1 kg of unroasted coffee beans were provided by a Regional Coffee Growers Cooperative, all belonging to the *Coffea arabica* category but from different subgroups.^18^ Upon receipt, the coffee samples were stored in a light-protected environment under controlled temperature conditions. Table S1 details the characteristics provided by the cooperative for each type of coffee analyzed. The percentage of impurities and moisture complies with the regulations of the Ministry of Agriculture, Livestock, and Supply (MAPA) for unroasted coffee beans.^18^

### 2.2 Coffee Roasting

In each experiment, approximately 8.2 ± 0.1 g of raw coffee beans were roasted in the laboratory. The heating program selected for roasting was based on a previous study by our group.^19^ A 1040 W Mini Retro electric popcorn popper, model RHP 310 (Nostalgia Products Group, Green Bay, WI, USA), was modified for this purpose. The main modifications included adding a potentiometer to the power cord, allowing control of the energy supplied to the popcorn popper’s electric heating system, and separating the power supply from the ventilation system, which is responsible for rotating the beans.

Based on previously obtained results,^19^ wherein it was investigated the impact of roasting on VOC associated with changes in the coffee aroma and the potential impact of analyzing the samples several days after roasting, this study adopted an intermediate roasting program with a total heating time of 8 min, where the power increased from 70% to 100% after the first 2 min.

### 2.3 Extraction

The extraction of volatile and semivolatile organic compounds was performed using dynamic headspace (DHS) sampling with 5 mm-OD glass thermal desorption (TD) tubes (Part No. 2414-1025) packed with a sorbent phase of Tenax TA and Carboxen (GL Sciences B.V. – Eindhoven, The Netherlands). The TD tubes were preconditioned according to the manufacturer’s recommendations. Additional information regarding the extraction performance of commonly used sorbent materials in TD tubes is available elsewhere for interested readers.^20–23^

A sampling port was added to the popcorn popper by replacing the original lid with a custom 3D-printed cover, as shown in Figure 1. This modification ensured an insulated environment so that the VOCs produced during roasting could be carried through an opening in the lid and be sorbed by the liner used. Additionally, the temperature was controlled using a TC4S module with a Pt100 probe (Autonics – Sao Paulo, SP, Brazil).

**Figure 1:**
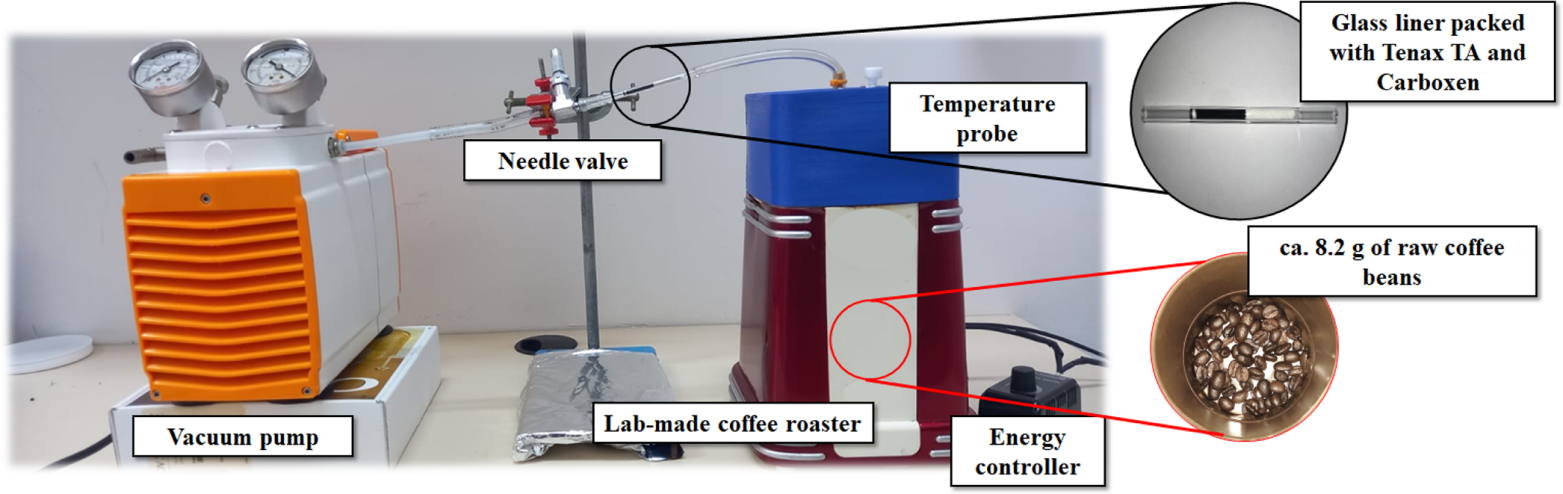
Experimental setup used for roasting raw coffee and capturing volatile (VOC) and semivolatile organic compounds (SVOC) produced during the process. Analyte sampling and preconcentration was attained using dynamic headspace (DHS) extraction.

Three distinct roasting stages were monitored during the 8 min roasting of raw coffee beans. The first monitored stage occurred from 0 to 2:40 min (time 1), the second from 2:40 to 5:20 min (time 2), and the third from 5:20 to 8:00 min (time 3). At the end of each stage, the liner was replaced with another preconditioned liner, totaling three liners per roasting. All liners were sealed immediately after removal from the system and were only opened when introduced into the OPTIC-4 multimode injector (GL Sciences B.V., Eindhoven, The Netherlands) for the thermal desorption step in the gas chromatograph.

As shown in Figure 1, a setup consisting of a vacuum pump, a needle valve, and inert Tygon S3 hoses (Saint Gobain) was used to generate a constant flow of 100 mL/min, exiting the roaster and passing through the liner with the sorbent phase. Each of the five available raw coffee samples was roasted in duplicate, resulting in a total of 30 measurements.

### 2.4 TD-GC×GC-TOFMS

Analyte introduction was performed using thermal desorption. After sampling, the TD tube was placed in the OPTIC-4 module. Analyte desorption was achieved using a fast-heating rate from 40 to 260 °C at 20 °C/s. The injection modes were evaluated in split and splitless conditions. The optimized method was performed using a 2 min splitless program. Helium was used as the carrier gas at a constant flow rate of 1.0 mL/min.

GC×GC-TOFMS analyses were conducted on an Agilent 8890 gas chromatograph coupled with a Pegasus BT 4D time-of-flight mass spectrometer (LECO Corporation – St. Joseph, MI, USA). Modulation was performed using a consumable-free QUADJET thermal modulator. For further information on modulation techniques, please read^24,25^ . The chromatographic columns consisted of a first-dimension (^1^D) column of 30 m × 0.25 mm × 0.25 μm Rxi-5MS and a second-dimension (^2^D) column of 2.2 m × 0.15 mm × 0.15 μm Rxi-17Sil MS (Restek Corporation – Bellefonte, PA, USA). The ^2^D column was housed in a secondary GC oven.

The 1D oven was heated from 50 to 260 °C at 4 °C/min, with a final isotherm of 5 min. The 2D oven and the modulator were programmed with offsets of +5 °C and +20 °C above the primary oven, respectively. The modulation period was 5 s.

The transfer line to the TOFMS and the ion source were maintained at 275 and 250 °C, respectively. Electron ionization at 70 eV was used in all experiments. The acquisition rate was 100 spectra/s with a mass range of 50–600 *m*/*z*.

### 2.5 Identification

GC Image software (Zoex – Houston, TX, USA) was used for the tentative identification of analytes through mass spectrum similarity searches using the NIST20 MS library (National Institute of Standards – Gaithersburg, MD, USA). LTPRI (linear temperature-programmed retention index) filtering was also utilized in the analyte identification process. Identification was performed using a minimum similarity of 75% between the experimental mass spectra and those in the NIST library, with a deviation of ±50 LTPRI units from the NIST values. Odor descriptors were obtained from the Good Scents Company database, which offers olfactometry data from pure compounds.^1,2,26^

Peak detection was performed using GC Image. Feature assignment was conducted using template matching based on marker peaks and chemical logic for chromatographic alignment.^27,28^

### 2.6 Data Processing

The MATLAB R2021b environment (MathWorks – Natick, MA, USA) was used for chemometric analysis. The processed peak tables were exported from GC Image in “.csv” format and concatenated to form a matrix **X**, containing all independent variables for modeling. The data set comprised 169 features from 30 roasted coffee measurements. The matrix **X** (30 × 169) was preprocessed using autoscaling in PLS Toolbox 9.0 (eigenvector Research Inc. – Wenatchee, WA, USA).

Data processing using PLS-DA allowed correlation of the area of volatile organic compounds in the measurements with each of the three studied roasting stages (property of interest). In this study, values of 1 were assigned to measurements from the first roasting stage (time 1), values of 2 to measurements from the middle stage (time 2), and values of 3 to measurements from the final stage (time 3). Thus, a vector representing the property of interest *y* (30 × 1) was formed.

The model for correlating experimental data (**X**) with the property of interest (*y*) was constructed, and the number of latent variables was selected based on the analysis of mean calibration and cross-validation errors (Venetian blind method). The model’s classification performance was evaluated using a confusion matrix, considering the values of true positive (TP), true negative (TN), false positive (FP), and false negative (FN).^29^

Permutation tests were used to help identify an overfit model as well as to determine whether the given model was significantly different from one built under the same conditions but with random data. If the modeling conditions were overfitting, they would often produce a fit to random data that was better than expected. Permutation tests were performed using PLS Toolbox and confirmed the absence of overfitting (Supporting Information). In general, values less than 0.05 indicate model significance at the 95% confidence level.

## 3 Results and Discussion

### 3.1 TD-GC×GC-TOFMS

The change in coffee bean color, transitioning from a greenish hue to a brownish one throughout the roasting process, is the most distinct change that can be observed during this stage. However, it is not only the color of the coffee beans that undergoes alterations during roasting. This change is accompanied by evolutions in the volatile profile of the beans due to a range of chemical reactions promoted by heating, including Maillard reactions, Strecker degradation, caramelization, degradation of chlorogenic acids, and lipid oxidation.^4^

The chromatograms obtained for the coffee samples at roasting times 1 and 3 (Figure 2A,B) reveal an evolution in the volatile profile of the coffee beans as the roasting time progresses. Additionally, the chromatograms of all 30 measurements indicate the presence of approximately 169 volatile compounds, which were trapped by the liners during roasting and tentatively identified. The analytes, as presented in Table 1, include aromatic hydrocarbons, alcohols, ketones, linear and branched hydrocarbons, nitrogenous compounds, esters, carboxylic acids, aldehydes, terpenes, and terpenoids, among others, consistent with previous studies.^1,2,4^ Odor descriptors for each compound were obtained from refs 1,2,26 . A blank run of the setup used in the study was conducted to eliminate any potential memory effects.

**Figure 2:**
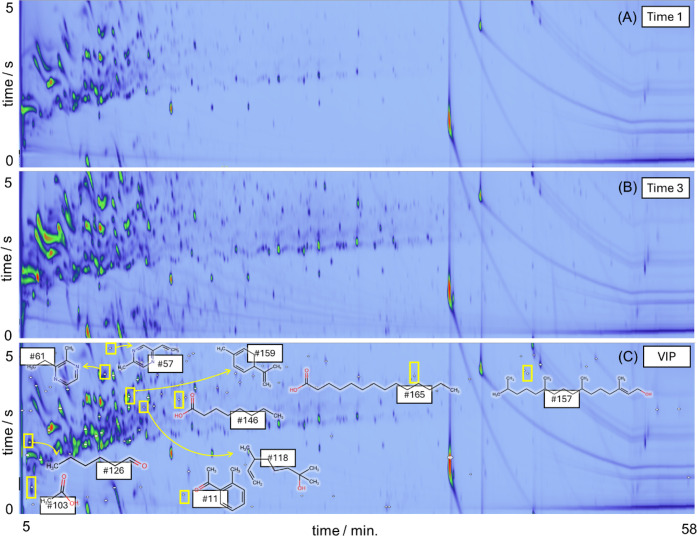
Examples of GC×GC-TOFMS chromatograms for different roasting time intervals of a raw coffee sample using DHS with a mixed sorbent phase (Tenax TA and Carboxen). (A) Time 1; (B) Time 3. The chromatogram at Time 1 corresponds to volatiles produced between 0 and 2:40 min, while at Time 3, it represents volatiles produced between 5:20 and 8:00 min. (C) Chromatogram of a coffee sample, highlighting (white dots) the 99 features identified using the VIP values approach in the PLS-DA model. Additionally, the chemical structures of some selected VIP compounds were also depicted in the figure.

**Table 1:** Analytes Identified in the Volatile and Semivolatile Fraction of Coffee Samples throughout the Roasting Process^a^

	Analyte identity	M.F.	ID Level	CAS	Odor	VIP value
1	(1-butylheptyl) benzene	C_17_H_28_	3	4537–15–9	-	1.60
2	(1-butyloctyl)-benzene	C_18_H_30_	2	2719–63–3	-	
3	(1-methylpropyl)-benzene	C_10_H_14_	3	135–98–8	-	
4	(1-methylpropyl)-cyclohexane	C_10_H_20_	2	758–01–7	-	1.31
5	(1-propylnonyl)-benzene	C_18_H_30_	2	2719–64–4	-	1.93
6	(2-methylbutyl) cyclohexane	C_11_H_22_	3	54105–77–0	-	
7	(*Z*)-14-methyl-8-hexadecen-1-ol	C_17_H_34_O_2_	3	-	-	1.47
8	1-(2-ethylphenyl) ethanol	C_10_H_14_O	3	-	-	1.50
9	1-(2-furanyl)-ethanone	C_6_H_6_O_2_	3	1192–62–7	powerful balsamic-sweet odor with a tobacco-like, almost narcotic pungency and floral undertones of balsamic-cinnamic character	
10	1-(2-methyl-1*H*-pyrrol-3-yl) ethanone	C_7_H_9_NO	3	-	-	1.77
11	1-(2-methylphenyl)-ethanone	C_9_H_10_O	2	577–16–2	sweet, hawthorn powdery, anisic, coumarinic, phenolic, burnt	1.01
12	1-(4-*tert*-butylphenyl) propan-2-one	C_13_H_18_O	3	81561–77–5	-	1.13
13	1-(acetyloxy)-2-propanone	C_5_H_8_O_3_	2	592–20–1	fruity-buttery, somewhat sour	1.89
14	1-(hydroxyphenyl)-2-methylpropane	C_10_H_14_O	3	-	-	
15	1-(methoxymethyl)-4-methylnaphthalene	C_13_H_14_O	3	71235–76–2	-	
16	1,1-dimethyl-2-propyl-cyclohexane	C_11_H_22_	3	81983–71–3	-	
17	1,2,3,4-tetrahydro-5-methyl-naphthalene	C_11_H_14_	2	2809–64–5	-	
18	1,2,3,4-tetrahydro-naphthalene	C_10_H_12_	2	119–64–2	-	1.76
19	1,2,3,4-tetramethyl-benzene	C_10_H_14_	3	488–23–3	-	1.76
20	1,2,3,5,6,7-hexahydro-1,1,2,3,3-pentamethyl-4*H*-inden-4-one	C_14_H_22_O	2	33704–61–9	diffusive, spicy, musk-like with strong floral note	
21	1,2,3-trimethyl-(1α,2α,3β)-cyclohexane	C_9_H_18_	2	7667–55–2	-	
22	1,2,3-trimethyl-benzene	C_9_H_12_	2	526–73–8	-	
23	1,2,4,5-tetramethyl-benzene	C_10_H_14_	2	95–93–2	weak, hazelnut	1.36
24	1,2,4-trimethyl-3-nitrobicyclo[3.3.1]nonan-9-one	C_12_H_19_NO_3_	3	129967–65–3	-	1.50
25	1,2,4-trimethylbenzene	C_9_H_12_	2	95–63–6	-	
26	1,2-dimethyl-benzene	C_8_H_10_	3	95–47–6	geranium	1.45
27	1,3,5-trimethylbenzene	C_9_H_12_	3	108–67–8	-	1.28
28	1,3-bis(1,1-dimethylethyl)-benzene	C_14_H_22_	2	1014–60–4	-	2.30
29	1,3-diethyl-5-methyl-benzene	C_11_H_16_	2	2050–24–0	-	1.14
30	1,7-dimethyl-naphthalene	C_12_H_12_	2	575–37–1	-	1.08
31	16-oxokahweol	C_22_H_28_O_4_	2	81760–47–6	-	2.03
32	1-butyl-cyclohexene	C_10_H_18_	2	3282–53–9	-	
33	1-ethyl-2,3-dimethylcyclohexane	C_10_H_20_	3	7058–05–1	-	1.18
34	1-ethyl-2-methyl-benzene	C_9_H_12_	2	611–14–3	-	
35	1-ethyl-2-methyl-cyclohexane	C_9_H_18_	2	3728–54–9	-	1.58
36	1-ethyl-3-methyl-benzene	C_9_H_12_	2	620–14–4	-	
37	1-ethylidene-1*H*-indene	C_11_H_10_	2	2471–83–2	-	1.02
38	1-isopropyl-2,3-dimethylcyclopentane	C_10_H_20_	3	489–20–3	-	
39	1-methyl-2-propyl-cyclohexane	C_10_H_20_	2	4291–79–6	-	
40	1-methyl-3-propyl-benzene	C_10_H_14_	2	1074–43–7	-	
41	1-methyl-3-propyl-cyclohexane	C_10_H_20_	3	4291–80–9	-	2.16
42	1-methyl-4-(1-methylbutyl)-cyclohexane	C_12_H_24_	2	54411–00–6	-	
43	1-methyl-4-(2-methyloxiranyl)-7-oxabicyclo [4.1.0] heptane	C_10_H_16_O_2_	2	96–08–2	mentholic	
44	1-methyl-4-propyl-benzene	C_10_H_14_	2	1074–55–1	-	
45	1-methyl-naphthalene	C_11_H_10_	2	90–12–0	naphthyl, chemical, medicinal, camphoreous	1.26
46	1-tetradecene	C_14_H_28_	3	1120–36–1	-	2.00
47	2,2-bis[5-(1,4-benzodithiafulven-6-yl)-2-thienyl]propane	C_27_H_20_S_6_	3	-	-	1.38
48	2,3-dihydro-1,3-dimethyl-1*H*-indene	C_11_H_14_	3	4175–53–5	-	1.38
49	2,3-dihydro-1,6-dimethyl-1*H*-indene	C_11_H_14_	3	17059–48–2	-	1.06
50	2,3-dihydro-4,7-dimethyl-1*H*-indene	C_11_H_14_	3	6682–71–9	-	1.32
51	2,3-dihydro-5-methyl-1*H*-indene	C_10_H_12_	2	874–35–1	-	1.04
52	2,4-decadienal	C_10_H_16_O	2	25152–84–5	orange-like, sweet and fresh-citrusy, fried, oily	
53	2,4-dimethyl-1-heptene	C_9_H_18_	2	19549–87–2	-	1.56
54	2,5-dimethyl-pyrazine	C_6_H_8_N_2_	2	123–32–0	roasted, nutty, grassy, “cornnuts”	
55	2,6-dibutyl-1,4-benzoquinone	C_14_H_20_O_2_	3	-	-	1.13
56	2,9-dimethyl-decane	C_12_H_26_	3	1002–17–1	-	
57	2-ethenyl-5-methyl-pyrazine	C_7_H_8_N_2_	2	13925–08–1	coffee	1.32
58	2-ethyl-1,3-dimethylcyclohexane	C_10_H_20_	3	7045–67–2	-	
59	2-ethyl-1,4-dimethyl-benzene	C_10_H_14_	2	1758–88–9	-	1.42
60	2-ethyl-1*H*-isoindole-1,3(2*H*)-dithione	C_10_H_9_NS_2_	3	35373–06–9	-	2.09
61	2-ethyl-3-methyl-pyrazine	C_7_H_10_N_2_	3	15707–23–0	nutty, earthy, must, bread, peanut	1.67
62	2-ethyl-6-methyl-pyrazine	C_7_H_10_N_2_	2	13925–03–6	roasted potato	
63	2-ethylhexyl salicylate	C_15_H_22_O_3_	2	118–60–5	orchid, sweet, balsam	
64	2-furancarboxyaldehyde	C_5_H_4_O_2_	3	98–01–1	sweet, woody, baked bread, caramel-like	1.14
65	2-furanmethanol	C_5_H_6_O_2_	2	98–00–0	slightly caramellic, warm-oily (well correlated with the underirable burnt and bitter note of dark-roasted coffees)	1.04
66	2-hexyldec-1-en-3-yne	C_16_H_28_	3	69520–29–2	-	1.17
67	2-methyl-tetrahydro-furan-3-one	C_5_H_8_O_2_	2	3188–00–9	sweet, brown, bready, nutty	1.89
68	2-methyl-3-buten-2-ol acetate	C_7_H_12_O_2_	2	24509–88–4	-	1.39
69	2-methyl-decane	C_11_H_24_	2	6975–98–0	-	1.00
70	2-methyl-propanoic acid 1-(1,1-dimethylethyl)-2-methyl-1,3-propanediyl ester	C_16_H_30_O_4_	3	74381–40–1	-	1.60
71	2-methyl-propanoic acid 3-hydroxy-2,4,4-trimethylpentyl ester	C_12_H_24_O_3_	3	74367–34–3	-	
72	2-nonadecanone	C_19_H_38_O	2	629–66–3	-	1.27
73	2-nonenal	C_9_H_16_O	2	18829–56–6	green, cucumber, aldehydic, fatty, citrus	
74	2-propenylidene-cyclobutene	C_7_H_8_	3	52097–85–5	-	1.44
75	2-*syn*-methyl-decalin	C_11_H_20_	2	2958–76–1	-	1.31
76	2-tetradecyloxirane	C_16_H_32_O	3	7320–37–8	-	
77	2-undecenoic acid	C_11_H_20_O_2_	3	15790–94–0	-	
78	3,4,5-trichloro-6-methylcyclohepta-2,4,6-trien-1-one	C_8_H_5_Cl_3_O_2_	3	-	-	1.54
79	3,5-dimethyl-octane	C_10_H_22_	2	15869–93–9	-	1.70
89	3,6-dimethyl octane	C_10_H_22_	2	15869–94–0	-	
81	3,7-dihydro-1,3,7-trimethyl-1*H*-purine-2,6-dione	C_8_H_10_N_4_O_2_	3	58–08–2	odorless	1.03
82	3,7-dimethyl-nonane	C_11_H_24_	2	17302–32–8	-	
83	3-ethylbicyclo [4.4.0] decane	C_12_H_22_	3	-	-	
84	3-methyl-decane	C_11_H_24_	2	13151–34–3	-	
85	3-methyl-nonane	C_10_H_22_	3	5911–04–6	-	
86	3-tetradecyne	C_14_H_26_	2	60212–32–0	-	
87	4,5-dimethyl-nonane	C_11_H_24_	3	17302–23–7	-	
88	4-methyl-nonane	C_10_H_22_	2	17301–94–9	-	1.85
89	4-*tert*-butyl-cyclohexanol acetate	C_12_H_22_O_2_	2	10411–92–4	fresh, fruity, floral, pine	
90	4-*tert*-butylcyclohexyl acetate	C_12_H_22_O_2_	2	32210–23–4	woody, floral, oily, fruity, herbal	1.09
91	5-(4-methylphenoxy) methyl-2-amino-1,3,4-thiadiazoles	C_10_H_11_N_3_OS	3	-	-	1.59
92	5-ethyl-2-methyl-octane	C_11_H_24_	3	62016–18–6	-	1.10
93	5-methyl-2(3*H*)-furanone	C_5_H_6_O_2_	2	591–12–8	sweet, coconut, tobacco and coumarin-like nuances	1.78
94	5-methyl-2-furancarboxaldehyde -	C_6_H_6_O_2_	2	620–02–0	sweet-spicy, warm, slightly caramellic	
95	6-ethyl-2-methyl-octane	C_11_H_24_	3	62016–19–7	-	
96	7,9-di*tert*-butyl-1-oxaspiro(4,5)deca-6,9-diene-2,8-dione	C_17_H_24_O_3_	2	82304–66–3	-	1.02
97	7-tetradecyne	C_14_H_26_	2	35216–11–6	-	
98	8-methyl-1-decene	C_11_H_22_	2	61142–79–8	-	2.34
99	9,12-octadecadienoic acid	C_18_H_32_O_2_	3	60–33–3	faint fatty	
100	9,12-octadecadienoic acid, methyl ester	C_19_H_34_O_2_	2	2462–85–3	-	1.08
101	9-octadecenoic acid	C_18_H_34_O_2_	3	112–80–1	fatty, waxy, lard, fried, tallow	1.53
102	acetate 2-furanmethanol	C_7_H_8_O_3_	2	623–17–6	sweet, green banana peel, estery	
103	acetic acid	C_2_H_4_O_2_	3	64–19–7	pungent, stinging sour	1.41
104	acetophenone	C_8_H_8_O	2	98–86–2	sweet, mimosa, acacia, floral, almond	
105	amberonne (isomer)	C_16_H_26_O	2	-	-	1.00
106	amberonne (isomer)	C_16_H_26_O	2	-	-	1.38
107	benzenemethanol	C_7_H_8_O	2	100–51–6	light floral, rose, phenolic, balsamic	1.12
108	bicyclo [4.1.0] heptan-2-one	C_7_H_10_O	3	5771–58–4	-	
109	bicyclo [5.3.0] decane	C_10_H_18_	2	16189–46–1	-	
110	biphenyl	C_12_H_10_	2	92–52–4	metallic, cinnamon-like, bergamot- and neroli-like	1.23
111	camphor	C_10_H_16_O	2	464–48–2	camphoreous	
112	cyclohexanone	C_6_H_10_O	3	108–94–1	minty, acetone	
113	decahydro-1,6-dimethyl-naphthalene	C_12_H_22_	3	1750–51–2	-	1.33
114	decahydro-*trans*-naphthalene	C_10_H_18_	2	493–02–7	-	1.04
115	decanal	C_10_H_20_O	3	112–31–2	sweet, waxy, orange peel, citrus, floral	1.02
116	diacetate 1,2-ethanediol	C_6_H_10_O_4_	3	111–55–7	green floral estery alcoholic	
117	diethyl phthalate	C_12_H_14_O_4_	2	84–66–2	odorless	
118	dihydromyrcenol	C_10_H_20_O	2	18479–58–8	fresh, lime cologne, herbal, air marine	1.46
119	dodecanal	C_12_H_24_O_3_	2	112–54–9	soap, waxy, aldehydic, citrus, green, floral	1.31
120	dodecanamide	C_12_H_25_NO	3	1120–16–7	-	2.40
121	eicosanoic acid methyl ester	C_21_H_42_O_2_	2	1120–28–1	-	1.24
122	ethylbenzene	C_8_H_10_	2	100–41–4	-	1.54
123	ethyl-pyrazine	C_6_H_8_N_2_	2	13925–00–3	peanut, butter, musty, woody, roasted, cocoa	1.11
124	heptane, 4-methyl-	C_8_H_18_	3	589–53–7	-	
125	hexadecanoic acid, methyl ester	C_17_H_34_O_2_	3	112–39–0	oily, waxy, fatty, orris	1.30
126	hexanal	C_6_H_12_O	2	66–25–1	very powerful, penetrating, fatty-green, grassy. Cut grass and unripe fruit	2.80
127	hexane, 3-ethyl-2,5-dimethyl-	C_10_H_22_	2	52897–04–8	-	
128	homosalate	C_16_H_22_O_3_	2	118–56–9	mild menthol	
129	indane	C_9_H_10_	2	496–11–7	-	1.04
130	isobutyl ether	C_8_H_18_O	3	628–55–7	-	
131	isopropyl myristate	C_17_H_34_O_2_	3	110–27–0	faint, oily, fatty	1.53
132	levomenthol	C_10_H_20_O	2	2216–51–5	peppermint, spicy, cooling, camphoreous	1.47
133	lilial	C_14_H_20_O	2	80–54–6	fresh, floral, lilac, powdery	1.21
134	limonene	C_10_H_16_	2	138–86–3	citrusy, lemon-like, fresh and sweet odor	
135	linalool oxide	C_10_H_18_O_2_	2	60047–17–8	powerful sweet-woody, penetrating odor with floral-woody-earthy undetones	1.31
136	longifolene	C_15_H_24_	2	475–20–7	sweet, woody, rose, medicinal	1.15
137	methyl (2-pyrimidyl) amine	C_5_H_7_N_3_	3	931–61–3	-	1.98
138	methyldecahydronaphthalene	C_11_H_20_	3	2958–75–0	-	1.04
139	methyl-pyrazine	C_5_H_6_N_2_	2	109–08–0	nuttty, cocoa, roasted, coffee, chocolate	
140	naphthalene	C_10_H_8_	2	91–20–3	pungent, choking dry-tarry odor of moderate to poor tenacity	1.06
141	nonanal	C_9_H_18_O	3	124–19–6	strong, soap-like, metallic	1.92
142	octadecanoic acid	C_18_H_36_O_2_	2	57–11–4	odorless, mild, fatty, waxy	1.56
143	octahydro-1*H*-indene	C_9_H_16_	2	496–10–6	-	1.15
144	octahydro-1*H*-indene	C_9_H_16_	2	3296–50–2	-	
145	octahydro-2,2,4,4,7,7-hexamethyl-1*H*-indene	C_15_H_28_	3	54832–83–6	-	1.49
146	octanoic acid	C_8_H_16_O_2_	2	124–07–2	oily rancid, sweat-like, repulsive even in dilution	1.35
147	*o*-*tert*-butyl cyclohexyl acetate	C_12_H_22_O_2_	2	88–41–5	fruit, woody, green, apple, herbal	1.55
148	*p*-cymene	C_10_H_14_	2	99–87–6	typical kerosene-like odor in high cocnentration	
149	pentadecanal	C_15_H_30_O	2	2765–11–9	fresh, waxy	
150	pentanoic acid, 2,2,4-trimethyl-3-hydroxy-, isobutyl ester	C_12_H_24_O_3_	3	244074–78–0	-	1.06
151	pentanoic acid, 3-mehtyl	C_6_H_12_O_2_	3	105–43–1	animalic, sharp acidic, cheesy, green with a fruit swweaty nuance	
152	pentyl-cyclohexane	C_11_H_22_	3	4292–92–6	-	
153	phenylacetaldehyde	C_8_H_8_O	2	122–78–1	green, floral, and sweet	
154	phenylmethane	C_7_H_8_	2	108–88–3	sweet-gassy odor, milder than that of benzene	1.81
155	phthalic acid, bis(2-ethylhexyl) ester	C_24_H_38_O_4_	2	117–81–7	-	
156	phthalic acid, isobutyl 3-methylbutyl ester	C_17_H_24_O_4_	2	-	-	
157	phytol	C_20_H_40_O	2	253686–88–3	-	1.14
158	phytone	C_18_H_36_O	2	502–69–2	oily, herbal, jasmin, celery, woody	1.29
159	*p*-mentha-1,5,8-triene	C_10_H_14_	2	21195–59–5	roasted	1.49
160	propyl- benzene	C_9_H_12_	2	103–65–1	-	
161	propyl-cyclohexane	C_9_H_18_	2	1678–92–8	-	
162	protoanemonine	C_5_H_4_O_2_	3	108–28–1	-	1.20
163	pseudocumene	C_9_H_12_	3	95–63–6	-	
164	tetrachloroethylene	C_2_Cl_4_	2	127–18–4	-	1.28
165	tetradecanoic acid	C_14_H_28_O_2_	2	544–63–8	waxy, fatty, soapy, coconut	1.38
166	thiofanox sulfone	C_9_H_18_N_2_O_4_S	3	39184–59–3	-	
167	*trans*-13-octadecenoic acid	C_18_H_34_O_2_	2	693–71–0	-	1.60
168	undecanal	C_11_H_22_O	2	112–44–7	waxy, aldehydic, citrus, watermelon	1.93
169	α-hexyl-cinnamaldehyde	C_15_H_20_O	2	101–86–0	-	

a Linear temperature programming retention indices (LTPRI) were obtained from NIST. All analytes were identified considering a minimum mass spectrum similarity of 75% and deviations less than ± 50 LTPRI units from the NIST Web Book. Analytes identified by the VIP values approach (Section 3.2) in the volatile and semivolatile fraction of coffee samples during the roasting procedure are also highlighted. Note: VOCs are listed in alphabetical order. ID Level: Description available at https://doi.org/10.1007/s11306-007-0082-2.

To ensure that all chemical information obtained by DHS-GC×GC-TOFMS is adequately interpreted, it is essential to employ appropriate data processing approaches, such as chemometrics. This allows patterns among the samples to be recognized, preventing the loss of any relevant information in the study.

### 3.2 Data Processing

A peak table-based approach was adopted, enabling robust statistical and multivariate analysis with reduced computational demands. Feature assignment utilized smart templates based on marker peaks and chemical logic for chromatographic alignment.^27,28^ More specifically, this workflow defined peak regions to establish chromatographic windows where analytes were consistently detected and matched using spectral information across chromatograms, generating comprehensive chemical profiles. In the current study, data processing was performed using PLS-DA, a widely employed approach in supervised analyses. If the generated model is successful, it will have calculated a threshold value to differentiate and classify the samples among the three present classes (class 1 – time 1; class 2 – time 2; and class 3 – time 3).

One of the initial steps in constructing the model was determining the number of latent variables (LVs) to be used. The chosen number of LVs corresponds to the dimensions of the subspace in which the original data will be projected. The selection of the number of LVs was based on the mean classification errors for cross-validation (Venetian blind). In this scenario, a PLS-DA model with 3 latent variables was built.

The confusion matrix of the PLS-DA model (Table S2) assists in identifying that the VIP (Variable Importance in Projection) values of the model contain information related to each of the three modeled roasting stages (i.e., three classes) and, consequently, to compounds that underwent concentration variations throughout roasting. Since the primary objective was to identify VIP scores through the PLS-DA model, permutation tests were used to evaluate overfitting. As shown in Table S3, such results indicated the absence of an overfitted PLS-DA model. In this context, the VIP values for each class were exported from the model, and those above the value of 1 were selected. As a result, 99 VIPs were identified, referring to variables (compounds) from the data matrix that are statistically significant for classifying samples into each class.

Therefore, 99 organic compounds among all the analytes trapped in the liners and analyzed by DHS-GC×GC-TOFMS exhibited statistically significant changes in their profiles during the roasting of raw coffee beans. All 99 compounds identified by this approach are highlighted in the last column of Table 1, and their respective chromatographic peaks can be visualized in the chromatogram presented in Figure 2C.

After listing all compounds using the VIP values approach, they were grouped into chemical classes, including aromatic hydrocarbons, alcohols, ketones, terpenoids, terpenes, hydrocarbons, carboxylic acids, nitrogen-containing compounds, esters, aldehydes, and purine. Figure 3 illustrates the changes in the areas of these chemical classes over the three monitored roasting stages (time 1, time 2, and time 3).

**Figure 3:**
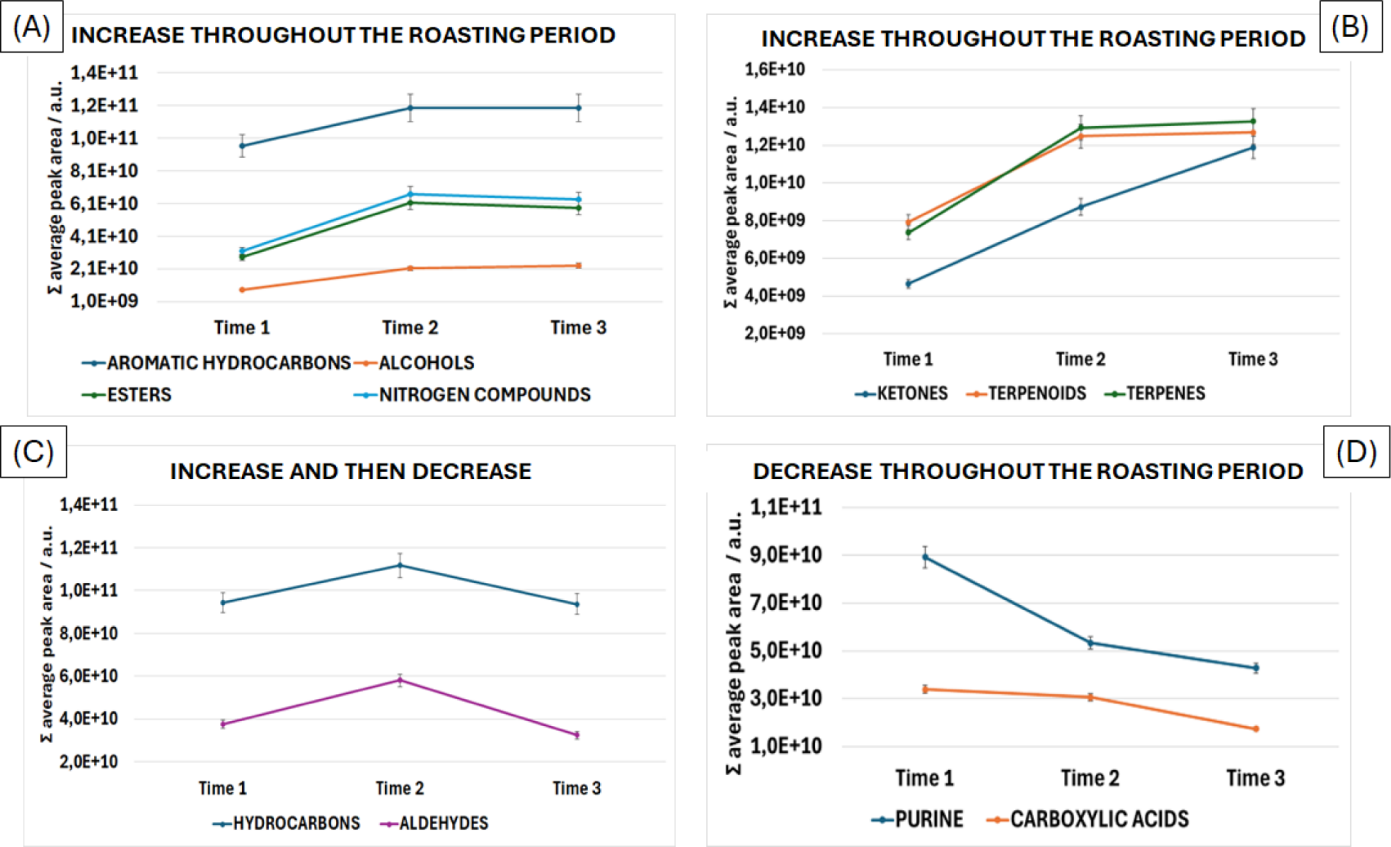
Roasting profiles of selected VOCs and SVOCs, grouped by chemical classes. Feature selection was based on the VIP variable selection approach. Different profiles can be identified: (A, B) analytes that show an increasing peak area during roasting; (C) intermediary analytes; and (D) carboxylic acids and purines, which exhibit a continuous decrease in peak area.

The development of coffee aroma and taste by roasting remains a highly empirical and handcrafted activity. In this scenario, the development of online approaches exploring mass spectrometric techniques to follow and obtain real-time information along the roasting process is highly desirable. Some of these researchers used online chemical ionization^30,31^ and photoionization,^16,32,33^ mass spectrometry, while others developed interesting setups to study the complex network of chemical changes that occur in a single bean.^7,14,15^ Moreover, an intriguing approach dynamically profiled coffee volatiles in the roasting plume exiting the roaster hood using a styrene-divinylbenzene resin (XAD-2).^10^ The interested reader is directed elsewhere for other studies in changes in volatilomics caused by roasting of food matrices.^34–40^

During the roasting step, the coffee bean can be seen as a pressurized reactor^4^ in which some of the reactions first observed by the chemist Louis-Camille Maillard in 1912 ^1^ can occur. The reactions bearing his name (Maillard reactions) involve the reaction between reducing sugars and amino acids or low-molecular-weight peptides to form several compounds. As proposed by Nursten et al.,^41^ Maillard reaction products may include (a) “simple” sugar dehydration/fragmentation compounds (furans, pyrones, cyclopentenes, carbonyl compounds, and acids); (b) “simple” amino acid degradation products (aldehydes and sulfur-containing molecules); and (c) volatiles produced by a cascade of further interactions (pyrroles, pyridines, imidazoles, pyrazines, oxazoles, thiazoles, and compounds from aldol condensations).

Among the four principal aliphatic acids (formic, acetic, glycolic, and lactic) that may be generated from glucose and fructose fragmentation,^2,42^ acetic acid (#103, Figure 2C) was successfully trapped and detected in this study. As with the other carboxylic acids (Figure 3D), this pungent compound exhibited a decreasing evolutionary profile along the roasting time. The decrease in acetic acid content, due to degradation or evaporation, during further roasting has been previously observed.^2^ Additionally, dark-roasted arabicas are prone to exhibit less acidity, with bitterness becoming the major taste.^1,2^ Tetradecanoic acid (#165) and octanoic acid (#146), both depicted in Figure 2C, were also found in other studies, the latter being found in Colombian, Santos, and green Mexican *arabica* coffees and in a commercial coffee powder, exhibiting an oily rancid and sweat-like odor.^1^ #165 peak profile evolution over the course of the roasting procedure is illustrated in Figure 4A. Its decreasing trend over time follows the general pattern observed for the carboxylic acid group. Additionally, the oleic acid (#101) and stearic acid (#142) ratio was already mentioned as a possible indicator of *robusta* in coffee blends.^2^ However, the present study identified a different distribution profile between these two free fatty acids, with stearic acid exhibiting a greater proportion than oleic acid instead of an equivalent (*arabica*) or smaller (*robusta*), as previously reported for the coffee samples they analyzed.

**Figure 4:**
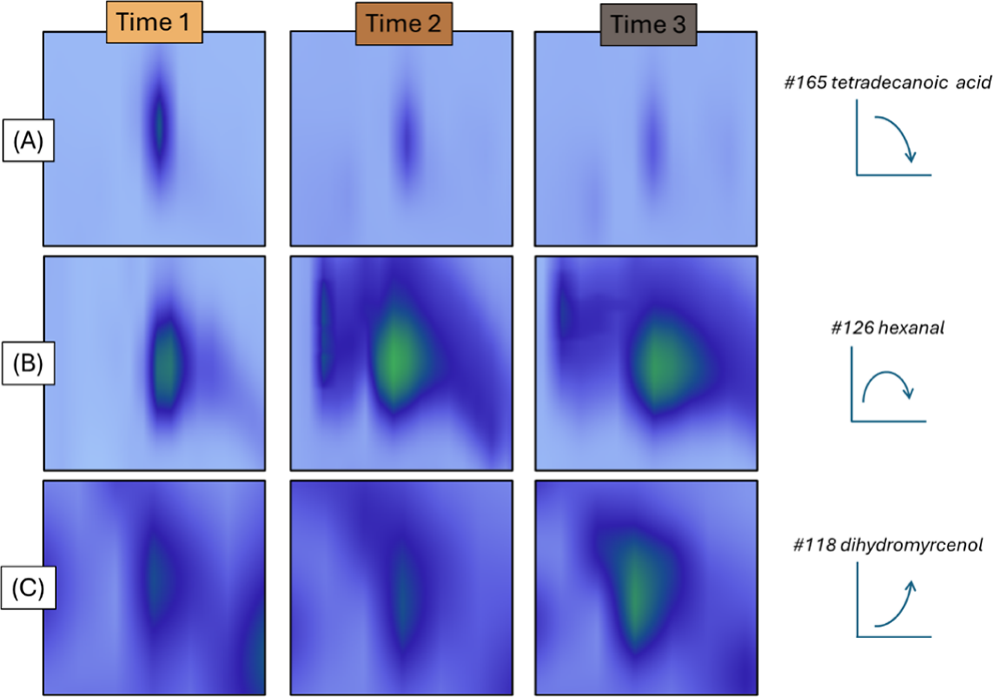
Distinct peak profiles of relevant compounds identified at each roasting stage. (A) Tetradecanoic acid, which exhibits a continuous decrease in peak intensity over the course of the roasting process; (B) hexanal, which increases initially and then slightly decreases; and (C) dihydromyrcenol, showing a continuous increase throughout the roasting experiment.

As part of the advanced stage of the Maillard reaction network,^43^ the Strecker degradation has preeminent importance in the formation of the coffee aroma spectrum. It involves the reaction between dicarbonyl compounds and amino acids to form a variety of products, including aldehydes, alkylpyrazines, aminoketones, carbon dioxide, and ketones.^1,2,4,43^ Ketones represent an important class of VOCs for coffee aroma. In general, they may represent 21.5% of the whole fraction of compounds that constitute the “coffee aroma”.^1^ Among them, many cyclic ketones, such as the VIP-selected (#11) 1-(2-methylphenyl)-ethanone, Figure 2C, may have their origin traced from carbohydrate dehydration/fragmentation.^1,2^ Imparting a coumarinic, phenolic, and burnt aroma in coffee samples, #11 and the other VIP-selected ketones with their interesting aroma descriptors, such as 1-(2-furanyl)-ethanone, (#9, tobacco-like odor, almost narcotic pungency, and floral undertones) and acetophenone (#104, mimosa, acacia, and floral odors), more than double their total amount throughout the roasting stages (Figure 3B).

Maillard reaction also provides important sulfur-containing chemicals for coffee aroma, such as thiazoles and thiols.^4^ Their key role in the flavors of roasted and cooked foods has been recognized for years.^1^ The present experimental setup could successfully trap and detect other sulfur-containing volatile compounds, such as dithione (#60) and the thiodiazole derivative (#91). Unfortunately, none of their odor descriptors could be found; however, it is well-known that sulfur volatiles are essential for the characteristic impact of roasted coffee, with at least 100 compounds being identified in coffee.^1,2^ Apart from Maillard reactions, their origin may also be associated with natural metabolic pathways of the coffee plant and storage of the roasted bean.^1^

Pyrazines constitute another important chemical family derived from the Maillard reaction network. As mentioned for the sulfur-containing compounds, pyrazines are important contributors to the flavor of raw, roasted, and cooked foodstuffs,^1^ and there are a few approaches developed to obtain them not only from natural sources (e.g., extraction and concentration) but also by developing and exploring modern biotechnology processes.^44^ The initial model experiments tried to elucidate pyrazine synthesis mechanisms through reactions between α-amino acids, carbohydrates, and small oxygen-containing molecules.^1^ All these movements aim to cope with the increasing demand for adding these nitrogen-containing compounds to foods. We have observed that the onset of roasting is accompanied by an increase in pyrazine concentration. This is directly related to the coffee’s aroma profile, as several pyrazines contribute to roasted and coffee-like notes, among others. Among the six pyrazines trapped and identified in this study, 2,5-dimethylpyrazine (#54) (roasted and nutty odors) is used in breakfast cereals, and 2-ethyl-3-methylpyrazine (#61, Figure 2C) (musty, earthy, bread, and peanut odors) is used in peanut products, popcorn, and bread.^44^ Considering the three VIP-selected pyrazines (#57, #61, and #123), all of them have already been previously reported in coffee samples.^1,2^ Apart from different roasting conditions and being a *robusta* coffee sample, 2-ethenyl-5-methylpyrazine (#57, Figure 2C) (coffee odor) exhibited a similar evolutionary profile as observed by Silwar and Lüllmann.^45^ Its peak area increased along the roasting process (from stage 1 to 2) and then remained almost the same until the end of the roasting period. Grouping the pyrazines with other VIP-selected nitrogen-containing compounds, they exhibited the evolutionary profile of initial growth and then their total area value remained almost constant until the end of the experiment (Figure 3A).

Aldehydes were one of the chemical groups that presented an evolutionary behavior of an initial rise followed by a gradual decline, Figure 3C. Considering the roasting scenario, one of their origins may be traced to the oxidative degradation of amino acids along their interaction with sugars at elevated temperatures.^1^ Another established route to aldehyde formation is through the autoxidation of unsaturated fatty acids.^1^ Considering the recognized presence of these lipids in coffee,^1^ it was interesting to not only visually observe a few very small oily droplets on the roasted bean surface at the end of the roasting period but also detect some unsaturated fatty acids in the current study (such as linoleic acid (#99), oleic acid (#101), and trans-13-octadecenoic acid (#167)). Some aldehydes mentioned in the literature that have their origin traced to linoleic acid and methyl linoleate autoxidation include hexanal (#126, Figure 2C) and 2,4-decadienal (#52). Besides being identified in the roasted product, the VIP-selected hexanal can be found in raw coffee beans and be involved in the staling process of roasted coffee beans when stored in an oxygen-containing atmosphere.^1^ Like the other aldehydes, hexanal also exhibited a general peak profile evolution of initial increase followed by a slightly decline—as illustrated in Figure 4B. With its characteristic green odor notes, 2-nonenal (#73) formation is also linoleic acid-related, being mentioned as a compound that helps balance some flavor notes, such as acidic, sour, and caramel.^1^

Even though they exhibited a constant increase profile along the roasting period monitored (Figure 3A), apparently, in general, low-molecular-weight alcohols do not tend to be potent odorants in coffee.^1^ Some exceptions may include alcohols from the recognized organoleptically active class of the isoprenoids (i.e., terpenoids). Five of these compounds were detected in the roasting process, with all of them (#31, #118, #132, #135, and #157) being statistically significant for sample classification in the PLS-DA model, since they increased steadily across the three observed roasting stages. Being possibly derived from linalool (already cited as an important odorant for roasted powder of *arabica* coffee^1^) oxidation, linalool oxide (#135) odor description is cited as powerful sweet-woody and a penetrating odor with floral-woody-earthy undertones. #135 was also found in strongly roasted coffee samples and in a raw coffee characterized by a “Rio” off-flavor.^1,46^ Levomenthol (#132, peppermint and spicy odors) and dihydromyrcenol (#118, Figure 2C, fresh, herbal, and air marine odors) were other interesting alcohol isoprenoids found in the roasted coffee samples. The steadily increasing profile of #118 during the coffee roasting experiment, in line with the trend observed for its chemical class, is shown in Figure 4C. Apart from not finding any odor description for it, 16-oxokahweol (#31) was another terpene-derived compound found in the coffee samples. This diterpene alcohol ester may be formed by the esterification of the hydroxyl group at the C-16 position of kahweol. And both diterpenes are cited as present in the lipid fraction of robusta and arabica coffees.^1^ In fact, kahweol is cited as very sensitive to acids, heat, and light, being reported as an unstable compound when in its purified form,^1,47^ which could possibly justify its absence in the DHS-trapped fractions.

Similarly to the alcohols, the terpenes’ evolutionary profile had a prominent increase in the first half of the roasting period, and then their total area value remained almost constant until the end of the roasting period (Figure 3B). Besides not being a VIP, the monoterpenic compound limonene (#134, citrussy, lemon-like, and fresh) was already widely found in coffee analysis. Being studied as a major contributor to the aroma of specialty coffees,^48,49^ for example. Among the other two VIP-selected terpenic compounds, an interesting one is *p*-mentha-1,5,8-triene (#159, Figure 2C), which is characterized by a roasted odor.

Although aliphatic and aromatic hydrocarbons are among the most abundant chemical families in the analyzed coffee samples, they are generally not considered to have strong odoriferous properties compared to other characteristic coffee constituents mentioned above.^1^ As shown in Table 1, little information is available regarding the odor descriptors of these compound classes. Nevertheless, they may play an indirect role in the aroma and flavor of coffee.

Obtaining such an extensive and rich data set on the identities and behaviors of coffee compounds during the roasting process of *Coffea arabica* represents a significant step toward a comprehensive understanding of the art of coffee roasting. Based solely on the sensory notes of the compounds listed in Table 1, it may be possible to achieve a coffee with more floral notes by roasting it somewhere between Times 2 and 3, as this time frame exhibits a higher concentration of compounds associated with these sensory descriptors. Similarly, for those seeking a more traditional coffee profile, roasting up to time frame 2 could be a viable option, as this stage presents the highest concentration of pyrazines, which contribute to roasted and coffee-like odor descriptors. It is important to emphasize that these observations are general insights based on chemical and sensory data, without delving into the complex antagonistic and synergistic interactions among compounds—key variables in this field.

Finally, developing new approaches that integrate chemical analysis and chemometric tools can help bridge existing gaps in coffee research. Moreover, combining such methodologies with additional data sources, such as sensory evaluations, has the potential to enhance coffee quality and refine roasting profiles. This, in turn, can be tailored to factors such as the quality of raw coffee, its origin, and brewing methods.

## 4 Conclusions

This paper introduces a novel setup and workflow by combining DHS-GC×GC-TOFMS and chemometrics to gain deeper insights into the evolutionary profiles of chemical compounds during the roasting stages of raw coffee beans. The study utilized a reliable experimental setup for real-time VOC and SVOC collection, using reusable and immobilized adsorbent phases for analyte sampling and preconcentration.

The enhanced resolving power of GC×GC-TOFMS was key to avoiding the loss of roasting-related chemical information due to peak overlap, as demonstrated by the complex volatile profiles obtained. Subsequently, PLS-DA combined with a VIP approach proved to be an effective method for studying the 99 organic compounds that underwent statistically significant concentration variations across the roasting stages, revealing distinct evolutionary profiles for each chemical family. In this scenario, the method developed proved to be suitable for monitoring the evolution of the coffee’s volatile profile during roasting.

Our findings undoubtedly contribute to a clearer understanding of the diverse and complex chemical transformations that occur during the roasting of raw coffee beans. This knowledge may help broaden the sensory spectrum of coffee by enabling personalized roast profiles, tailoring aroma and flavor to consumers’ preferences.

## Supplementary Material



## Notes

Information of the coffees used in the study and their respective characteristics (Table S1); confusion matrix for the PLS-DA model (Table S2); probability of model insignificance vs permuted samples (Table S3) (PDF)
