# Supplementary material for: Enhancing the Understanding of Aroma Formation during Coffee Roasting Using DHS-GC×GC-TOFMS and Chemometrics
Source: ACS Omega. 2025 Jun 13;10(25):26871–83. doi: 10.1021/acsomega.5c01783 (PMC12224095; doi:10.1021/acsomega.5c01783)
Supplement: Supplementary file 1 [file ao5c01783_si_001.pdf]

SUPPORTING INFORMATION FOR

Enhancing the understanding of aroma formation during coffee roasting using DHS-GC×GC-TOFMS and chemometrics

Andre Cunha Paiva,[1,2] Carlos Alberto Teixeira, [1,2] Leandro Wang Hantao[1,2,*]

[1]Instituto de Química, Universidade Estadual de Campinas, 270 Monteiro Lobato, Campinas, SP 13083-862, Brasil. E-mail address: wang@unicamp.br.

[2]Instituto Nacional de Ciência e Tecnologia (INCTBio), SP, Campinas, 13083-862, Brasil.

Supporting Information

1. COFFEE DESCRIPTION

Table S1. Information of the coffees used in the study and their respective characteristics. All the coffee samples have the state of Minas Gerais as their region of origin.

Sample	Impurity* / %	Moisture* / %	Coffee quality**
A	0.2	11.6	Strictly soft
B	0.3	11.6	Hard+
C	0.3	11.7	Fermented hard
D	0.4	10.8	2 riadas and 3 hard
E	0.3	11.6	2 riadas rio and 3 hard

* Impurities are below 1%, and moisture is below 12.5% according to MAPA regulations.
** Sensory classes based on the quality of *Arabica coffee* according to the official Brazilian classification *(1)*.

2. PLS-DA MODELING

Table S2. Confusion matrix for the PLS-DA model constructed with 3 LVs.

	Real class		
	**time 1**	**time 2**	**time 3**
**Predicted as time 1**	10	0	0
**Predicted as time 2**	0	10	0
**Predicted as time 3**	0	0	10
**Unassigned**	0	0	0

Table S3. Table summarizing the probability of model insignificance versus permuted samples for each Y-column, displaying results from Wilcoxon and Rand t-test for both self-prediction and cross-validation. Values less than 0.05 indicate model significance at the 95% confidence level.

Probability of Model Insignificance vs. Permuted Samples

Y-column: 1

	Wilcoxon	Rand t-test
Self-Prediction	0.001	0.005
Cross-Validated	0.000	0.005

Y-column: 2

	Wilcoxon	Rand t-test
Self-Prediction	0.014	0.045
Cross-Validated	0.001	0.008

Y-column: 3

	Wilcoxon	Rand t-test
Self-Prediction	0.040	0.037
Cross-Validated	0.001	0.005

3. REFERENCES

1. Brazil. *MAPA - MINISTRY OF AGRICULTURE, LIVESTOCK AND SUPPLY - Technical Regulation of Identity and Quality for the Classification of Processed Green Coffee Beans*. Brasilia, Diário Oficial da União. 2003.
